# The olivocochlear reflex strength in awake chinchillas is relevant for behavioural performance during visual selective attention with auditory distractors

**DOI:** 10.1038/s41598-020-71399-8

**Published:** 2020-09-10

**Authors:** Macarena Bowen, Gonzalo Terreros, Felipe N. Moreno-Gómez, Macarena Ipinza, Sergio Vicencio, Luis Robles, Paul H. Delano

**Affiliations:** 1 Departamento de Neurociencia, Facultad de Medicina, Universidad de Chile, Santiago, Chile; 2 Departamento de Otorrinolaringología, Hospital Clínico de la Universidad de Chile, Santiago, Chile; 3 Departamento de Fonoaudiología, Facultad de Medicina, Universidad de Chile, Santiago, Chile; 4 Instituto de Ciencias de la Salud, Universidad de O’Higgins, Rancagua, Chile; 5 Laboratorio de Bioacústica y Ecología del Comportamiento Animal, Departamento de Biología y Química, Facultad de Ciencias Básicas, Universidad Católica del Maule, Talca, Chile; 6 Biomedical Neuroscience Institute, BNI. Facultad de Medicina, Universidad de Chile, Santiago, Chile; 7 Programa de Fisiología y Biofísica, ICBM, Facultad de Medicina, Universidad de Chile, Santiago, Chile; 8 Centro Avanzado de Ingeniería Eléctrica y Electrónica, AC3E, Universidad Técnica Federico Santa María, Valparaíso, Chile

**Keywords:** Auditory system, Cognitive neuroscience, Sensory processing

## Abstract

The auditory efferent system comprises descending projections from the cerebral cortex to subcortical nuclei, reaching the cochlear receptor through olivocochlear fibres. One of the functions attributed to this corticofugal system is to suppress irrelevant sounds during selective attention to visual stimuli. Medial olivocochlear neurons can also be activated by sounds through a brainstem reflex circuit. Whether the individual variability of this reflex is related to the cognitive capacity to suppress auditory stimuli is still controversial. Here we propose that the individual strength per animal of the olivocochlear reflex is correlated with the ability to suppress auditory distractors during visual attention in awake chinchillas. The olivocochlear reflex was elicited with a contralateral broad-band noise at ~ 60 dB and ipsilateral distortion product otoacoustic emissions were obtained at different frequencies (1–8 kHz). Fourteen chinchillas were evaluated in a behavioural protocol of visual attention with broad-band noise and chinchilla vocalizations as auditory distractors. Results show that the behavioural performance was affected by both distractors and that the magnitudes of the olivocochlear reflex evaluated at multiple frequencies were relevant for behavioural performance during visual discrimination with auditory distractors. These results stress the ecological relevance of the olivocochlear system for suppressing natural distractors.

## Introduction

Attention is a cognitive function that works as a biological filter allowing us to select relevant stimuli from the environment and to partially ignore the rest^1^. Attention can be mediated by top-down and bottom-up neural mechanisms, depending on whether the cognitive process is driven by a voluntary goal or by the stimulus salience, respectively^2^. It has been proposed that top-down processing during selective attention modulates sensory responses at different levels of the nervous system maintaining task-directed behaviour^2,3^.

Cross-modal paradigms of visual selective attention with auditory distractors have demonstrated changes in neural activity at different levels of the auditory pathway, including cortical regions^4–6^, subcortical nuclei^7^, auditory nerve, and the cochlear receptor^8,9^. Previously, we demonstrated in chinchillas that selective attention to visual stimuli modulates the amplitude of auditory-nerve compound action potentials and cochlear microphonics, most likely through the activation of the auditory efferent system^9^.

The auditory efferent system is a neural network that comprises descending projections from the auditory cortex to the inferior colliculus, cochlear nucleus and superior olivary complex^10–12^, from where the medial olivocochlear (MOC) bundle emerges making synapses directly with outer hair cells of the cochlea^13^. The functionality of these corticofugal pathways has been demonstrated in bats, chinchillas and humans^14–17^, and these descending projections are proposed to be the mediators of selective attention top-down effects on the cochlear receptor^18,19^. In addition, peripheral modulations of the cochlear receptor during visual attention have been demonstrated in humans^20–22^. For example, Wittekindt et al. (2014) showed that during periods of visual attention the amplitude of distortion product otoacoustic emissions (DPOAE) was reduced whereas during auditory attention their amplitude remained unchanged.

Medial olivocochlear fibres can be activated by ipsilateral and contralateral acoustic stimulation (CAS), producing a suppressive effect on cochlear responses, that is highly variable among different individuals^23^. Previously, we showed that larger suppressions of auditory nerve responses by contralateral broad-band noise (BBN) are associated with better performance in a visual selective attention task with auditory distractors in mice^18^. However, in that work, the MOC reflex was evaluated in anesthetized mice, a condition that is known to underestimate olivocochlear effects^24,25^. Additionally, whether the correlations between the individual strength of the olivocochlear reflex and the cognitive capacity to avoid auditory distractors are frequency specific or greater with ecologically relevant auditory distractors is unknown.

**Figure 1:**
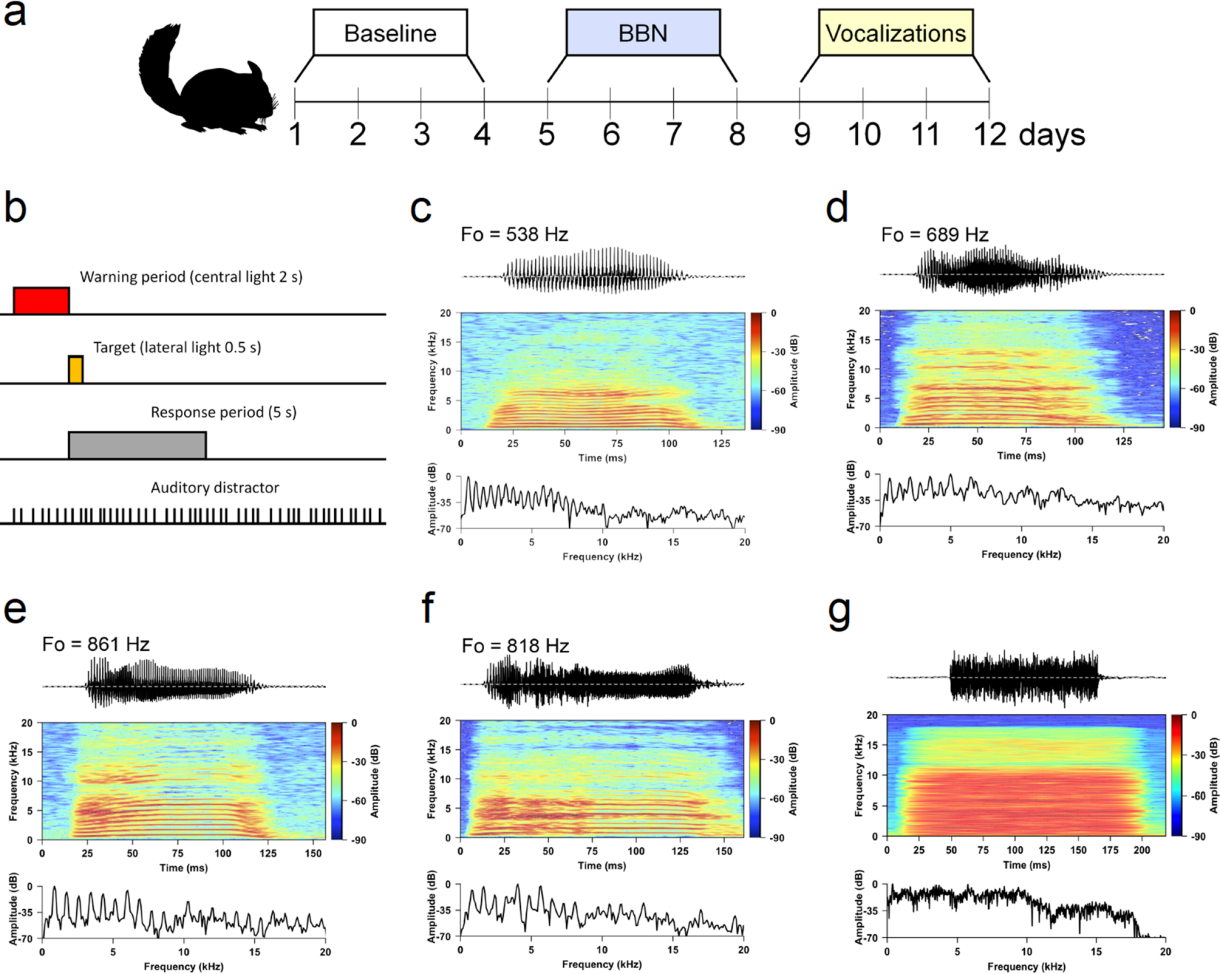
Behavioural paradigm and acoustic distractors. (**a**) Experimental protocol of twelve days. In the first four days chinchillas performed the visual discrimination without auditory distractors (baseline). Between days 5 and 9, a broad-band noise (BBN) was introduced as an auditory distractor, while between days 9 and 12, chinchilla distress vocalizations were used as distractors. (**b**) Temporal course of the visual discrimination task. (**c**–**f**) Acoustic characteristics of male chinchillas’ vocalizations. Panels **c**–**f** correspond to the order of presentation of vocalizations used from days 9 to 12 in the behavioural protocol. Information in the panel from top to bottom shows: fundamental frequency (F0), oscillogram, spectrogram, and spectral power measured in the middle of the pulse. (**g**) Broad-band noise used as auditory distractor between days 5 and 8.

Here, we measured the contralateral acoustic suppression of DPOAEs with broad-band noise in awake chinchillas and evaluated their behavioural performance in a visual selective attention task using two different auditory distractors: broad-band noise and chinchilla distress vocalizations (VOC)^26^ (Fig. 1). Our results indicate that the effects of contralateral noise on multiple DPOAE frequencies in awake chinchillas are relevant for predicting behavioural performance in a visual selective attention task with both types of auditory distractors.

## Results

### Behavioural performance with auditory distractors

**Figure 2:**
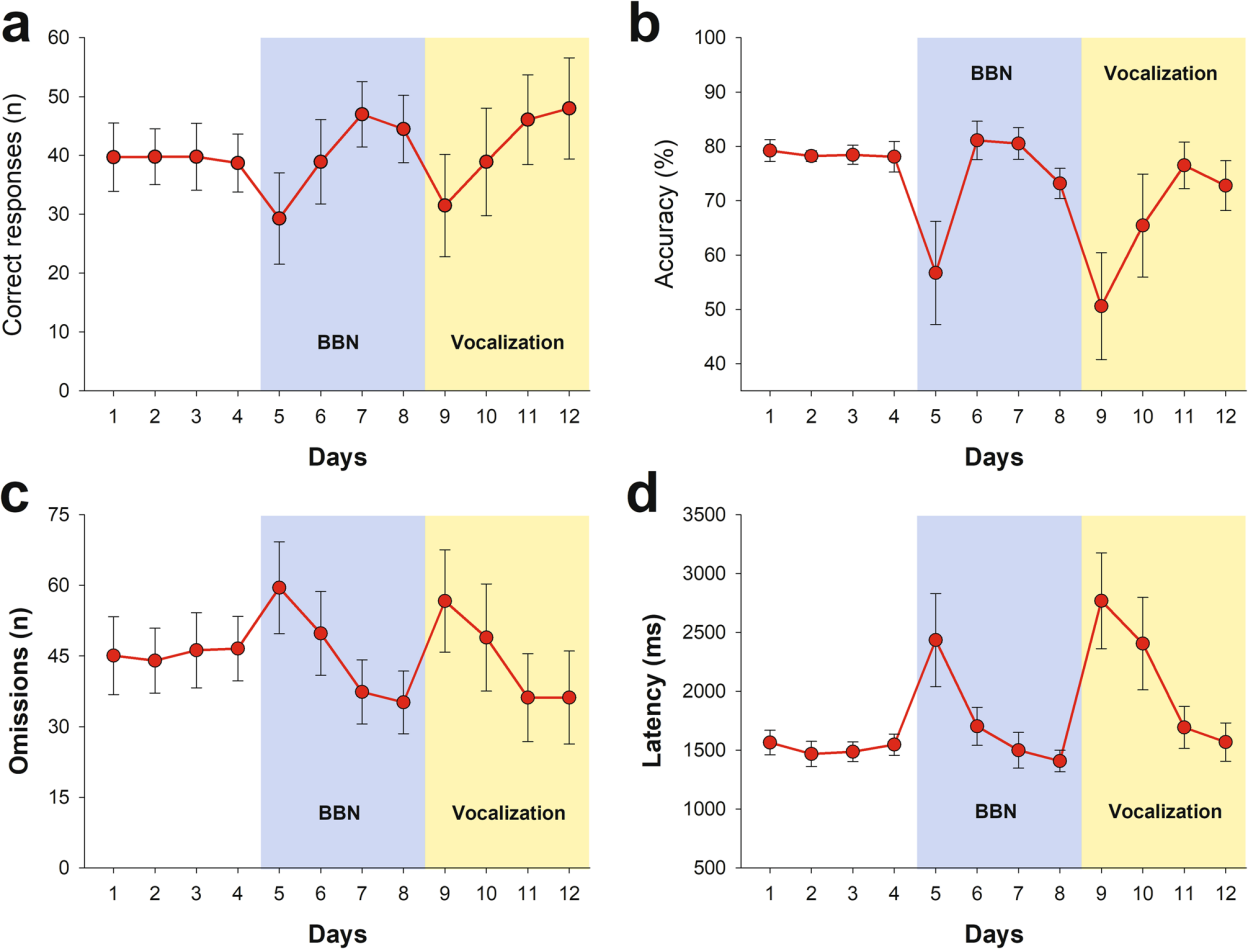
Grand average effects of auditory distractors on behavioural responses during the 12 days experimental protocol. (**a**) Correct responses. (**b**) Accuracy. (**c**) Omitted trials. (**d**) Mean latency of correct responses. We found significant effects for behavioural performance on days 5 and 9 (see results section in the main text). Data are shown as mean ± SEM (n = 14 chinchillas).

Figure 2 shows the effects of BBN and VOC in the behavioural performance of chinchillas (n = 14) during the 12 days of the experimental protocol, showing that there were significant effects in days 5 and 9. To facilitate the reading of the results, each day of the protocol will be referred using the acronym of the three stages (BL (for baseline), BBN and VOC) followed by the number 1 to 4, corresponding to the day of presentation (e.g.: day 1 = BL1, day 9 = VOC1). The generalized linear mixed effects models (GLMM) showed an effect of the stage of behavioural protocol on the number of correct responses (*X*^2^_(2)_ = 30.818, p < 0.0001) and omissions (*X*^*2*^_(2)_ = 38.528, p < 0.0001). For these two variables, significant a posteriori Tukey pairwise tests were found for the comparisons between BL1 and BBN1, and between BL1 and VOC1, but not between BBN1 and VOC1 (Table 1). For accuracy, there was also an effect of the stage of behavioural protocol (*X*^*2*^_(2)_ = 285.657, p < 0.0001), and all pairwise comparisons (BL1-BBN1, BL1-VOC1 and BBN1-VOC1) showed significant differences (Table 1). Regarding latency, the linear mixed effects models (LMM) analysis showed a significant effect of the stage of behavioural protocol (*X*^*2*^_(2)_ = 9.671, p = 0.008), where the only significant pairwise difference was obtained between BL1 and VOC1 latencies (Table 1). Consequently, the results shown in Fig. 2 and Table 1 evidence that both auditory distractors significantly disrupted the behavioural performance of chinchillas during the visual discrimination task.

**Table 1:** A posteriori Tukey pairwise comparisons for models comparing behavioural performance between days BL1 (day 1), BBN (day 5) and VOC1 (day 9).

Variable	Contrast (days)	p value
Correct	1–5	** < 0.0001**
	1–9	** < 0.0001**
	5–9	0.9958
Omissions	1–5	** < 0.0001**
	1–9	** < 0.0001**
	5–9	0.7497
Accuracy	1–5	** < 0.0001**
	1–9	** < 0.0001**
	5–9	**0.0003**
Latency	1–5	0.0922
	1–9	**0.0171**
	5–9	0.6548

Significant results (p < 0.05 are bolded).

### CAS effects on DPOAE

**Figure 3:**
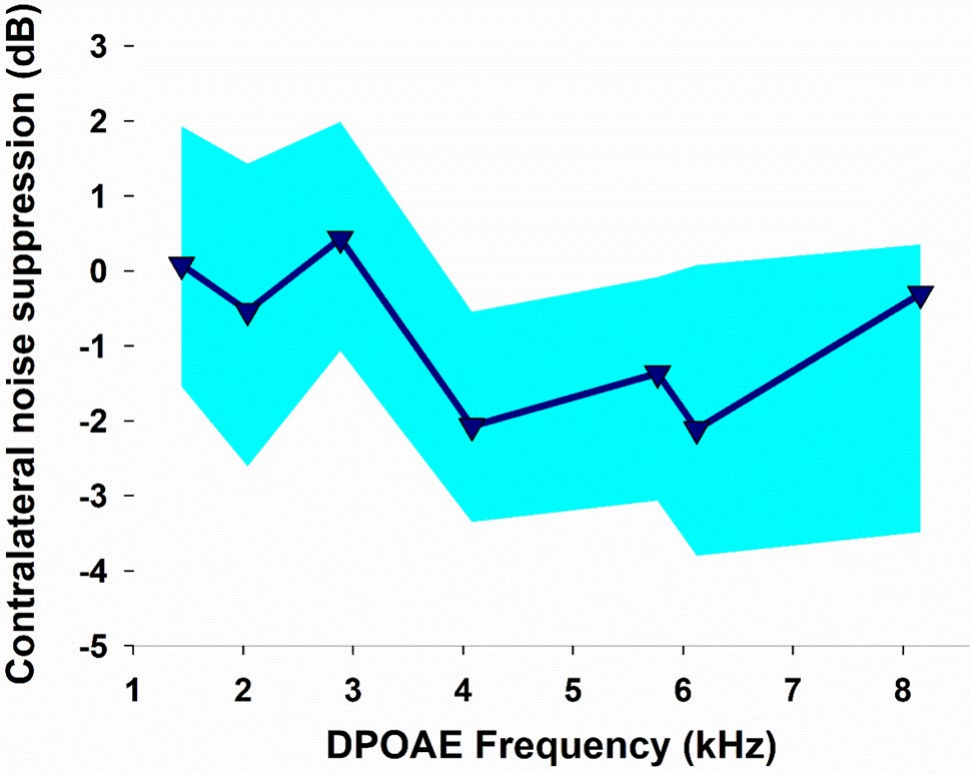
Contralateral noise suppression of DPOAEs at each of the seven evaluated frequencies in awake chinchillas (n = 13). The contralateral noise suppression of DPAOEs obtained at the different frequencies was largest between 4 and 6 kHz. Arrowheads point the seven frequencies used for DPOAE measurements. Data are shown as median values (triangles) while shaded areas correspond to the interquartile range (25–75%).

The MOC reflex strength is referred as the dB of change between the DPOAE amplitudes with and without CAS, a measure that was obtained before the behavioural protocol, with amplitude reductions as negative values. Animals were kept awake and unanaesthetised during all the DPOAE measurements, since evidence shows that the MOC reflex strength is underestimated in anesthetized chinchillas^25^. Figure 3 shows the effects of contralateral acoustic stimulation on DPOAE amplitudes for frequencies between 1 and 8 kHz. An average CAS effect of − 1.28 ± 1.75 dB (mean ± SEM) was obtained across frequencies, with larger effects at 4 kHz (− 2.16 ± 0.57 dB) and 6 kHz (− 2.05 ± 0.60 dB).

### Medial olivocochlear reflex strength and behavioural performance

Next, we studied whether the individual variability in the strength of contralateral acoustic suppression in awake chinchillas could be a predictor of behavioural performance in the visual task with auditory distractors. To evaluate the effect of each auditory distractor on the behavioural performance we chose the first day of presentation of the distractor as the measure of interest (BBN1 and VOC1), based on the daily results observed during the 12 days of experimental protocol (Fig. 2). Generalized linear models were used to assess whether the different DPOAE frequencies and the individual variability of the olivocochlear reflex strength could account for the individual behavioural performance. Models obtained by stepwise backward selection for broad-band noise (BBN1) show that the magnitudes of the olivocochlear reflex evaluated at multiple DPOAE frequencies are relevant for the number of correct responses, omissions and accuracy during the selective attention task (Table 2). In relation to the latency of correct responses, only two DPOAE frequencies (1,440 and 5,769 Hz) were relevant for visual discrimination during BBN1 (Table 2).

**Table 2:** Significance of CAS on DPOAE amplitude at different frequencies included in the final models for variables measured during BBN1.

Variable	DPOAE frequency	LR Chisq	Df	Pr(> Chisq)
Correct	1,440 Hz	193.032	1	** < 0.0001**
	2040 Hz	209.883	1	** < 0.0001**
	2,884 Hz	82.916	1	** < 0.0001**
	4,080 Hz	38.687	1	** < 0.0001**
	5,769 Hz	165.536	1	** < 0.0001**
	6,125 Hz	123.643	1	** < 0.0001**
	8,160 Hz	104.694	1	** < 0.0001**
Omissions	1,440 Hz	194.342	1	** < 0.0001**
	2040 Hz	217.588	1	** < 0.0001**
	5,769 Hz	210.693	1	** < 0.0001**
	6,125 Hz	75.509	1	** < 0.0001**
	8,160 Hz	99.504	1	** < 0.0001**
Accuracy	1,440 Hz	201.831	1	** < 0.0001**
	2040 Hz	125.730	1	** < 0.0001**
	2,884 Hz	190.658	1	** < 0.0001**
	4,080 Hz	24.102	1	** < 0.0001**
	5,769 Hz	202.865	1	** < 0.0001**
	6,125 Hz	173.071	1	** < 0.0001**
	8,160 Hz	104.458	1	** < 0.0001**
Latency	1,440 Hz	4.704	1	**0.0301**
	2040 Hz	3.125	1	0.0771
	2,884 Hz	0.837	1	0.3602
	4,080 Hz	2.458	1	0.1169
	5,769 Hz	5.011	1	**0.0252**
	6,125 Hz	1.778	1	0.1823
	8,160 Hz	1.250	1	0.2636

Significant results (p < 0.05 are bolded).

Models obtained by stepwise backward selection for vocalizations (VOC1) show that the magnitudes of the olivocochlear reflex evaluated at multiple DPOAE frequencies are significant for the number of correct responses, omissions and accuracy during day 9 of the behavioural protocol (VOC1) (Table 3). Importantly, in the case of VOC1 models for accuracy, omissions, and correct responses, the likelihood ratio Chi-square values were always highest for 4,080 Hz. In addition to that, the only significant frequency in the VOC1 model that involved the latency of correct responses was 4,080 Hz.

**Table 3:** Significance of CAS on DPOAE amplitude at different frequencies included in the final models for variables measured during VOC1.

Variable	DPOAE frequency	LR Chisq	Df	Pr(> Chisq)
Correct	2040 Hz	110.473	1	** < 0.0001**
	2,884 Hz	5.442	1	**0.0197**
	4,080 Hz	166.264	1	** < 0.0001**
	5,769 Hz	63.658	1	** < 0.0001**
	6,125 Hz	36.066	1	** < 0.0001**
	8,160 Hz	48.991	1	** < 0.0001**
Omissions	2040 Hz	48.695	1	** < 0.0001**
	2,884 Hz	4.530	1	**0.0333**
	4,080 Hz	107.106	1	** < 0.0001**
	5,769 Hz	21.056	1	** < 0.0001**
	6,125 Hz	31.216	1	** < 0.0001**
	8,160 Hz	23.398	1	** < 0.0001**
Accuracy	2040 Hz	134.077	1	** < 0.0001**
	2,884 Hz	7.939	1	**0.0048**
	4,080 Hz	145.124	1	** < 0.0001**
	5,769 Hz	43.438	1	** < 0.0001**
	6,125 Hz	11.396	1	**0.0007**
	8,160 Hz	53.240	1	** < 0.0001**
Latency	1,440 Hz	1.325	1	0.2496
	2040 Hz	1.883	1	0.1700
	2,884 Hz	0.520	1	0.4709
	4,080 Hz	11.968	1	**0.0005**
	5,769 Hz	3.491	1	0.0617
	6,125 Hz	2.653	1	0.1033
	8,160 Hz	1.357	1	0.2441

Significant results (p < 0.05 are bolded).

**Figure 4:**
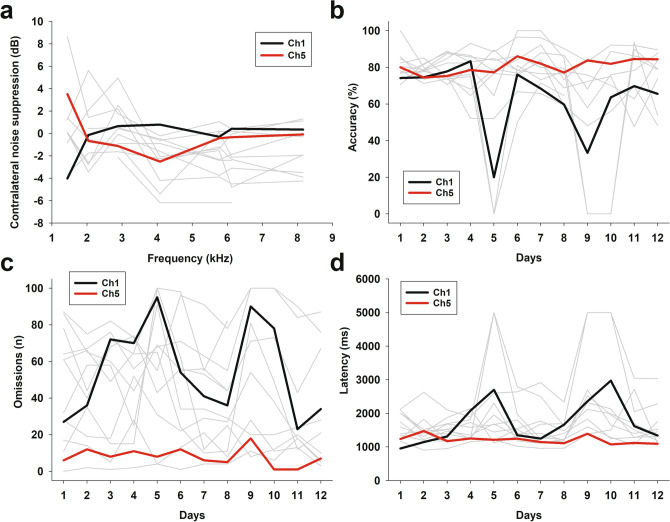
MOC reflex strength and behavioural performance at the individual level (**a**) Contralateral noise suppression of DPOAE at different frequencies. Gray traces show data for each animal, evidencing individual variability of the MOC reflex strength (n = 13). Traces for two chinchillas (Ch1, Ch5) are coloured as examples of: (i) an average MOC reflex (red traces), and (ii) an almost absent MOC reflex (black traces) at 4 kHz. (**b**–**d**) Individual performance (gray traces) during the behavioural protocol, showing (**b**) accuracy, (**c**) omissions, and (**d**) latency of correct responses. Note that the chinchilla with an almost absent reflex at 4 kHz (Ch1, black traces) was severely affected by auditory distractors, with reductions in accuracy, increased omissions and longer latencies during BBN1 and VOC1 (days 5 and 9). On the other hand, the chinchilla with an average MOC reflex strength at 4 kHz (Ch5, red traces) was barely affected by the auditory distractors.

Figure 4 shows data at the individual level, relating the MOC reflex strength with the behavioural performance. Two chinchillas are coloured as illustrative examples of: (i) an average MOC reflex at 4 kHz (red traces), and (ii) an almost absent MOC reflex (black traces). The chinchilla with the average MOC reflex was almost unaffected by the auditory distractors during the behavioural protocol, while the animal with almost absent MOC reflex at 4 kHz was severely affected by BBN and VOC.

## Discussion

Here we provide evidence that the effects of contralateral noise suppression on DPOAEs at multiple frequencies in awake chinchillas are significant predictors of the behavioural performance during a visual discrimination task in the presence of auditory distractors. This evidence suggests that the individual variability in the strength of the olivocochlear reflex is an important factor that aids in the ability to ignore auditory stimuli during visual selective attention.

Broad-band noise and chinchilla vocalizations produced a reduction in the number of correct responses and in the percentage of accuracy, and increased omissions in the visual selective attention task. Importantly, vocalizations produced a greater effect than BBN on accuracy, and they elicited a significant difference in the latency of correct responses as compared to baseline (days 1 and 9), while there was a non-significant latency difference between BL1 and BBN1 (Table 1). These results can be explained by the ecological importance of distress calls in chinchillas. While BBN is an anthropogenic stimulus, vocalizations are emitted by stressed chinchillas as an alarm call to other members^26,27^, hence they carry relevant social and semantic information for chinchillas^28^.

The distractive effects of BBN and VOC are evident for the first days of presentation in the behavioural protocol (BBN1; VOC1), but they tend to disappear from the second day of presentation of the auditory distractors. This could be explained by habituation to the distractors, which is a well-known neural mechanism, in which a novel stimulus is relevant for the individuals, while a repeated presentation of a stimulus generates habituation^29,30^. It is important to note that in our results, this effect is not generalized for the auditory modality, as after being habituated to BBN stimuli, the introduction of vocalizations in day 9, produced behavioural effects that were stronger than those induced by BBN in day 5. However, it might be possible that the behavioural effects of the vocalizations were underestimated due to the order of presentation of the auditory distractors. Future experiments could test whether inverting the order of presentation of auditory distractors (e.g. vocalizations presented in day 5, BBN in day 9) may produce larger effects with vocalizations.

The effects observed in chinchillas are different from those described in mice (see Fig. 2 from Terreros et al.^18^), in which a broad-band noise distractor did not reduce the number of correct responses and did not affect omissions. These differences could be explained by specific differences between species; mice and rats are more active animals than chinchillas. This behavioural difference is evident from the number of omissions these species present in a similar visual discrimination task: chinchillas omit ~ 45 out of a total of 110 trials (40.9%) (Fig. 2, present work) while mice omit ~ 30 out of a total of 110 trials (27.3%) (see Fig. 2 from^18^), and rats ~ 3 out of a total of 50 trials (6.0%)^31^. Comparing the results from mice^18^ and chinchillas, we can state that auditory stimuli presented during a visual selective attention task are more disruptive for the behavioural performance of chinchillas than for mice.

We evaluated contralateral noise suppression of DPAOEs for frequencies between 1 and 8 kHz in awake chinchillas, obtaining an average suppression of − 1.28 ± 1.75 dB across frequencies, while the largest average suppressions (~ 2 dB) were observed for frequencies between 4 and 6 kHz. Similarly, in a previous work we demonstrated that the contralateral suppression of auditory-nerve compound action potentials (CAP) in the chinchilla were largest for frequencies between 3 and 4 kHz^25^. However, in that work we found CAP suppressions reaching ~ 10 dB, a value that is similar to the CAP reductions of ~ 11 dB that were obtained by electrical stimulation of olivocochlear fibres in chinchillas^32^. These differences in the magnitude of the olivocochlear reflex effects are in agreement with previous results showing that the olivocochlear effects are larger when evaluated with neural responses than with otoacoustic emissions^33,34^.

Although previous experimental data indicate that the suppressive effects of contralateral sounds on cochlear responses are mediated by olivocochlear fibres^35^, it might be possible that the suppressive effects on DPOAEs could be partially produced by activation of the middle-ear reflex that also reduces cochlear sensitivity. However, to avoid this confounding effect, we used low-intensity (60 dB) contralateral broad-band noise, which is an unlikely elicitor of middle-ear muscle reflexes. Furthermore, in chinchillas, the suppressive effect of the middle-ear reflex is known to be limited to low frequencies (< 1 kHz), as after cutting the tendons of the middle ear muscles (tensor tympani and stapedius) the CAS effects on DPOAEs disappear for frequencies < 1 kHz, while for frequencies around 4.5 kHz they persist^36^.

In the work by Wolter and colleagues^36^, the magnitude of the effect of CAS on DPOAEs at 4.5 kHz was ~ 0.5 dB, while in our work we obtained average suppressions up to ~ 2 dB for 4 kHz (Fig. 3). One important difference is that they measured the olivocochlear reflex in anaesthetised chinchillas, while we did it in awake chinchillas. As mentioned above, previous evidence shows that the magnitude (strength) of the olivocochlear reflex is underestimated in anaesthetised guinea pigs, mice and chinchillas^24,25,37,38^, as the activity of MOC neurons is dependent on the level of anaesthesia^39^.

In a previous work in mice, we found a correlation between the number of correct and omitted trials with the strength of the MOC reflex on auditory nerve responses (wave I from auditory brainstem responses)^18^. In that work, MOC reflexes were obtained in anaesthetised mice, while in the present work the olivocochlear reflex was evaluated in awake chinchillas. In addition, as stated above there are species specific differences between the behavioural effects of auditory distractors on visual discrimination in mice and chinchillas. Despite these differences, we confirmed our previous findings in mice, that larger suppressions of auditory nerve responses by contralateral noise are associated to a better performance in a visual selective attention task with auditory distractors (Fig. 4). In the present work, the strength of the olivocochlear reflex evaluated with DPOAE at multiple positions of the cochlea (different DPOAE frequencies) are relevant for predicting correct and omitted responses during the first days with auditory distractors in chinchillas. Moreover, we found that the models for predicting behaviour during VOC1 presentation were more significant for frequencies of 4 kHz, as this frequency had the highest likelihood ratio Chi-square values. The DPOAE at 4,080 Hz corresponds to a middle position in the cochlea of chinchillas, a place where the majority of medial olivocochlear synapses on outer hair cells are found in this species^40^.

In humans, de Boer and Thornton^41^ showed that the individual magnitudes of suppression of click evoked otoacoustic emissions correlated with the learning skills in a speech-in-noise discrimination task, showing that the olivocochlear system played a relevant role in the acquisition of this task. However, more recent experiments performed by the same group using the same experimental paradigm produced contradictory results^42^. The new data seems to indicate that, in humans, the olivocochlear system may act in a dynamic (e.g., attention- or experience-dependent), rather than in a purely reflexive control of cochlear gain^42^.

The present results and previous evidence^8,9,18,21,22^ in animal models and humans show that the auditory efferent system aids in ignoring auditory distractors during visual selective attention tasks. However, there are several additional brain mechanisms that are essential for selective attention, including central gain control, feature selectivity and oscillatory interactions between different cortical areas^2, 43^. In this sense, the auditory efferent system should be considered as an additional mechanism that aids the brain to filter irrelevant responses before they reach the central nervous system.

In conclusion, we show that the individual variability of the olivocochlear reflex strength assessed at different DPOAE frequencies is important for the behavioural performance during a visual selective attention task in the presence of auditory distractors.

## Methods

### Animals and protocol

A total of 18 male chinchillas (*Chinchilla laniger*) weighing between 500 and 700 g were used at the start of the behavioural training. From these chinchillas, four were excluded: two of them stopped the training program due to health problems and the other two did not reach criteria in the behavioural protocol (see below). Fourteen chinchillas successfully finished the training program and were included in the protocol. All the chinchillas were housed in individual cages in a temperature and humidity-controlled room with an inverting dark/light cycle (lights on from 8 P.M. to 8 A.M.). During the experimental period, they were given *ad-libitum* access to water and were food deprived, maintaining 85 to 90% of their free-feeding weight. All procedures were approved by the local committee of Bioethics (Comite de Bioetica Animal, permit number #0844, Facultad de Medicina, Universidad de Chile) and made in accordance with the *Guidelines for the Care and Use of Laboratory Animals*^44^. Efforts were made to minimize the number of animals used and their suffering. The experiment was divided in three stages: (1) measurement of DPOAEs suppression by contralateral noise in awake animals, (2) behavioural training and (3) 12 days behavioural protocol. All of these experiments were performed in double-walled sound-attenuating rooms.

### Measurement of DPOAEs suppression by contralateral noise in awake chinchillas

The awake chinchillas were carefully placed in a soft body and neck restrictor, maintaining the room temperature at 23–24 °C and with lights off. Chinchillas bear this restriction for around 30–40 min, and movements were monitored with a video camera inside the acoustic chamber. The strength of the MOC reflex was evaluated by means of DPOAEs, measured at 2f1–f2, without and with contralateral acoustic stimulation (CAS) using broad-band noise. Vocalizations were not used in the measurements of the MOC reflex. DPOAEs test/re-test was measured in two different weeks in each animal, using a protocol of 1,440 trials equally divided in three blocks of 480 trials: before, during and after the contralateral acoustic stimulation. All experiments were controlled with custom-made programs developed in C language (LabWindows). One of the chinchillas was not able to finish this protocol; therefore, the strength of the OC reflex elicited with contralateral broad-band noise on DPOAEs was measured in 13 of the 14 chinchillas included in the behavioural protocol.

### Auditory stimuli

Seven frequencies were used as ipsilateral primary tones, delivered to the right ear, for eliciting DPOAEs at different positions of the cochlea (f2 = 1,440, 2040, 2,884, 4,080, 5,769, 6,125 and 8,160 Hz), while contralateral broad-band noise (~ 60 dB SPL) was delivered to the left ear. Both stimuli were digitally generated by two synchronized PCI boards (6,071-E, National Instruments) at 100,000 samples/s, attenuated with PA-5 programmable attenuators (System 3, Tucker-Davis Technologies) and delivered through ER-2 transducers (Etymotic Research) sealed to both external auditory meatus and pinna. Primary tones were presented at a rate of 4 Hz with a duration of 15 ms, 5 ms rise/fall time, a fixed ratio of *f2/f1* = 1.25 and *L1/L2* = 65/60 dB SPL, with a delay of 200 ms. Contralateral non-continuous broad-band noise (BBN, 0.2–10 kHz) was delivered at a presentation rate of 4 Hz with a duration of 170 ms. At the beginning of each experiment sound pressure level calibrations were made in both ears using an Etymotic^®^ microphone.

### Data acquisition and analysis

The resulting distortion products at 2f1–f2, were recorded with an ER-10B + microphone system (Etymotic Research) with 40 dB gain, amplified 10,000×, filtered between 0.1 and 10 kHz (Krohn-Hite, model 3,323), digitized at 40,000 samples/s and stored for off-line analysis. DPOAE amplitudes were calculated using Fast Fourier transforms during each of the three periods: baseline (without CAS), during CAS and recovery (without CAS). Contralateral noise effects were assessed by the change (reduction or increase) of DPOAE amplitudes in dB. Thus, MOC reflex strength was defined as [during CAS amplitude—baseline amplitude]. Recordings and off-line analyses were done by custom-made software written in C language (LabWindows).

### Behavioural apparatus and training procedures

Two experimenters blinded to the MOC reflex strength of each animal performed the behavioural procedures. All animals were trained during ~ 3 to 4 months, five days a week, in a two-choice visual discrimination task previously used by us in rats^31,45^, chinchillas^9^ and mice^18,46^. The behavioural task was performed in an operant mesh cage identical to the one used in Delano et al.^9^, located inside a double-walled sound-attenuating room. The front panel of the cage had a central light (neutral cue) above the food dispenser and two lateral lights (targets), located above the right and left lever^9^. Each trial began with the onset of the central light (warning period) for 2 s, followed by the onset of one (randomly) of the target lights for a period of 0.5 s. Chinchillas were trained to respond by pressing the corresponding lever below the lateral light during the response period of 5 s, from the onset of the target light. Intertrial time interval (ITI) period varied randomly between 27 and 33 s (Fig. 1). Correct responses during the response period, were rewarded with a 45 mg pellet (Noyes PJNI-0045 Chinchilla Food Pellet; Research Diets, New Brunswick, NJ). Incorrect responses (pressing the opposite lever during the response period), central light and ITI responses were punished with a 40 s timeout period, during which all lights were turned off. Trials in which chinchillas did not respond were defined as omissions and were not punished. The behavioural variables measured were accuracy (i.e., [correct responses/(correct responses + incorrect responses)] *100), number of correct, and incorrect responses, number of omitted trials and latency of lever-pressing in correct responses (time between the onset of the target light and the lever-pressing response).

During the training period the number of trials per session, target-light duration, ITI period and punishment time were progressively modified according to the animal performance. After chinchillas reached an accuracy of at least 70% during a session of 110 trials with protocol values of 0.5 s target-light duration, ITI of 27–33 s and punishment time of 40 s, they were recruited into the experimental protocol.

### Experimental protocol

The behavioural protocol was evaluated for 12 days divided into 3 stages of 4 days with 110 trials each (Fig. 1). In the first 4 days (baseline period), chinchillas performed the two-choice visual discrimination task without auditory distractors. During days 5–8, chinchillas did the visual discrimination task in the presence of broad-band noise as an auditory distractor and during days 9–12 in the presence of a male chinchilla vocalizations. Two of the fourteen chinchillas completed only 8 days of protocol including baseline and BBN as distractors and were not exposed to vocalizations.

### Auditory distractors

Two different auditory distractors were used during the behavioural protocol: (1) BBN (0.02–20 kHz), as an irrelevant distractor and (2) male chinchilla vocalizations, as an ecologically relevant distractor. All vocalizations used were previously recorded in a distress context for the study of Moreno-Gomez et al.^26^. We used four clean harmonic male vocalizations (one for each of the four days with VOC (days 9–12)) with the fundamental frequency (F0) between 538 and 861 Hz and dominant frequency around 1,200 Hz (Fig. 1). BBN and VOC distractors were presented binaurally at ~ 65 dB SPL through a speaker (Sony, frequency response 20–20,000 Hz) located 1 m above the operant cage in free field conditions. In order to diminish habituation, auditory distractors were delivered at an irregular rate centred at 2.5 ± 1.0 Hz (1.5–3.5 Hz, pseudo-randomly distributed).

### Data analysis

The effect of both auditory distractors on behavioural performance during the first days of each of the three stages of the behavioural protocol (days 1, 5 and 9) were evaluated using linear mixed effects models or generalized linear mixed effects models, where LMMs were used to analyse the latency of correct responses and GLMMs were used to analyse the number of correct responses (CORR), the number of omitted responses (OMI) and the accuracy of responses (ACCU). For CORR and OMI a Poisson family with a log link was used, and for ACCU a binomial family with a logit link was used. These procedures are appropriate for the analyses of count and proportional data, respectively^47^. Individual intercepts were included as a random effect in order to account for data dependence. The day (stage of the behavioural protocol) was incorporated as a categorical fixed effect and its significance was evaluated using Wald chi-square tests. Tukey *posteriori* pairwise comparisons were performed when the effect of day was significant. These analyses were performed using the R^48^ libraries lme^49^, car^50^, and emmeans^51^. The association between MOC reflex strength (DPOAE CAS-induced changes) and behavioural data for each stage of behavioural protocol by separate was evaluated using generalized linear models, where data were fitted using the same family distributions mentioned above. The full model contained the MOC reflex strengths measured at different frequencies. Model selection was performed using a stepwise backward model selection procedure using Akaike information criterion. The significance of the factors included in the final model was obtained using a type III deviance test. These analyses were performed using the R^48^ libraries MASS^52^ and car^50^.

## Data Availability

The datasets generated and analysed during the current study are available from the corresponding author on reasonable request.
